# Preliminary evaluation on the effect of oral omega-3 supplementation from herring caviar oil in primary open-angle glaucoma patients

**DOI:** 10.1007/s10792-025-03693-1

**Published:** 2025-07-21

**Authors:** Jingyi Luo, Shu Tu, Kaijing Li, Runcai Yang, Yixiu Lin, Jiayu Deng, Xiang Chen, Jian Ge

**Affiliations:** 1 State Key Laboratory of Ophthalmology, Guangdong Provincial Key Laboratory of Ophthalmology and Visual Science, Zhongshan Ophthalmic Center, Sun Yat-sen University, Guangzhou, 510000 Guangdong China; 2 Glaucoma Department, Zhongshan Ophthalmic Center, Sun Yat-sen University, Guangzhou, 510000 Guangdong China; 3 Guangdong Provincial Clinical Research Center for Ocular Diseases, Zhongshan Ophthalmic Center, Sun Yat-sen University, Guangzhou, 510000 Guangdong China

**Keywords:** Dietary supplementations, Omega-3 fatty acids, Herring caviar oil, Phospholipid, Primary open-angle glaucoma

## Abstract

**Background:**

The study aimed to assess the neuroprotective effect of phospholipid-rich omega-3 fatty acids from herring caviar oil in POAG patients with intraocular pressure (IOP) control.

**Methods:**

A single-center, observational, short-term, preliminary evaluation of three months was conducted. Fifty eyes of POAG patients with IOP were included and divided into the control group (*n* = 31) and the intervention group (*n* = 19) receiving one capsule of omega-3 fatty acids supplementation per day. All the participants underwent comprehensive clinical assessment at baseline and 3 months, including best-corrected visual acuity (BCVA), IOP, visual field (VF) test, optical coherence tomography (OCT). Primary outcomes were median deviation (MD) and pattern standard deviation (PSD) scores of VF, while secondary outcomes included BCVA, IOP, retinal nerve fiber layer thickness (RNFLT) of OCT, and adverse events.

**Results:**

At baseline, no significant differences were observed between the 50 patients in terms of age, sex, antiglaucomatous medications, BCVA, IOP, VF, or RNFLT. After three months, the intervention group exhibited a statistically significant improvement in MD value (*p* = 0.01). The change of PSD revealed a slightly greater reduction in the intervention group compared to controls, albeit with borderline significance (*p* = 0.08). A minor decrease in IOP was noted in the intervention group at three months, compared to the control group (*p* = 0.07). No significant differences were observed in other secondary outcomes, and no adverse events were reported.

**Conclusion:**

Oral omega-3 fatty acids supplementation derived from herring caviar oil is potentially neuroprotective and safe for IOP-controlled POAG patients, and it may serve as a clinically valuable additional option. However, these results necessitate confirmation with an appropriately designed randomized controlled study.

## Introduction

Glaucoma is the leading cause of irreversible blindness worldwide, characterized by progressive degeneration of retinal ganglion cells (RGCs) and impairment of visual function [1]. The mechanism of RGC degeneration is multifactorial. Elevated intraocular pressure (IOP) is the only modifiable risk factor for glaucoma, whereas glaucomatous optic neuropathy (GON) continuously progresses in some patients even though the IOP is controlled [1, 2].

Neuroprotective strategies, therefore, become an imperative research area by targeting IOP-independent mechanisms to inhibit or delay RGC death and promote neuro-regeneration pathways [3, 4]. Dietary supplementation emerges as a feasible neuroprotective intervention in which antioxidative nutrition is capable of inhibiting reactive oxygen species production and neuroinflammation in many ocular diseases [5–8].

Increasing studies have shown that essential fatty acid has neuroprotective effects on glaucoma [2, 5, 9–12]. The long-chain omega-3 polyunsaturated fatty acids mainly include docosahexaenoic acid (DHA), eicosapentaenoic acid (EPA), and alpha-linolenic acid (ALA), playing a central role in cell membrane fluidity, increased neurite outgrowth, neurotransmission, signal transduction, anti-inflammatory effect, and so on [13–16]. Nevertheless, humans cannot synthesize omega-3 fatty acids in vivo and must intake them from dietary supplementation.

Omega-3 fatty acids in phospholipid form is suggested to be more bioavailable than other forms [17]. Herring caviar oil from the Arctic Ocean contains phospholipid-rich omega-3 fatty acids relative to other commercial fish oil; however, its effect on POAG patients remains to be determined. Here, we conducted a preliminary clinical study to evaluate the effect of an oral omega-3 fatty acids supplementation from phospholipid-rich herring caviar oil on IOP-controlled POAG patients.

## Methods

### Study design and participants

This was a single-center, observational, short-term, preliminary study in IOP-controlled POAG patients over three months initiated by investigator (J.G.). Written informed consent was acquired from all participants enrolled in the Clinical Research Center of Zhongshan Ophthalmic Center (ZOC), Sun Yat-sen University, China, between January 2022 and June 2022. The study was approved by the ethics committee and institutional review board of ZOC and adhered to the tenets of the Declaration of Helsinki. The inclusion/exclusion criteria are listed in Table 1. All patients were diagnosed with POAG and under anti-glaucoma treatment for more than two years.

**Table 1:** Inclusion and exclusion criteria

Inclusion criteria	Exclusion criteria
1. Aged > 18 yrs	1. Atopy, allergic disorders, or allergic to the study supplemens and its excipients
2. Diagnosed with POAG*:	2. Glaucoma surgery less than 3 months
Adult onset	3. Previous other ocualr surgery
Optic disc or RNFL structural abnormalities	4. Ocular diseases other than POAG that could affect examination of VF and OCT
Reliable and reproducible VF abnormality	5. Usage of ocular medicines except IOP-lowering drops and lubricants
Open anterior chamber angle	6. Systemic diseases and general treatments
No history or findings of pseudoexfloiation, pigment dispersion, uveitis or other secondary glaucoma	7. Pregnant and lactant women
3. Average IOP ≤ 18 mm Hg with or without treatment	
4. Initial AGIS score = 6–20	
5. Informed consent	

*VF* visual field, *RNFL* retinal nerve fiber layer, *AGIS* advanced glaucoma intervention study

^*^The diagnosis criteria is according to the China Glaucoma Guideline (2020) and the POAG Preferred Practice Pattern developed by American Academic of Ophthalmology

Personal interviews were conducted with all subjects to obtain their general and ocular conditions, including hypertension, diabetes, cardiopathy, history of eye disease and surgery, and medication treatment. Prior to the baseline examination, participants were required to discontinue using nutritional supplements and other related medicines for at least two weeks. Participants could continue to use IOP-lowering medicines and were asked to strictly follow the recommendation from a senior ophthalmologist during the study.

A complete ophthalmological assessment was made at baseline, including best-corrected visual acuity (BCVA) using the early treatment diabetic retinopathy study (ETDRS) charts, IOP with a Goldmann applanation tonometer, slit-lamp biomicroscopy with gonioscopy, fundus examination using a hand-held 90-dioptre lens with slit-lamp and color fundus photography, VF performed by standard automated perimetry with the SITA standard 30–2 program (Humphery Field Analyzer 860, Carl Zeiss Meditech Inc., Dublin, CA, USA), and optical coherence tomography (OCT, DRI OCT Triton, Topcon Corporation, Tokyo, Japan) assessing the peripapillary retinal nerve fiber layer thickness (RNFLT). IOP measurements were performed three times and taken the average. The baseline and subsequent examinations took place between 9 a.m. and 11 a.m. The VF tests were regarded as reliable only if false responses and fixation losses were less than 33% [18]. Images of OCT were obtained when the image quality scores (IQS) were greater than 40 without artifacts (motion, defocus, segmentation error, decentration, blink, or masking), and all scanned images were manually checked by experienced ophthalmologists. If the results of VF test and OCT were unreliable or of poor quality, repeated examinations were performed, or the results were excluded otherwise. If both the patient’s eyes were eligible, one eye per patient was randomly selected.

For intervention group, the patients took one capsule of the ROMEGA supplementation (composition see Table 2; online source: http://www.romega.com.cn, June 2024) every day for three months; likewise the recruited patients in the control group without ROMEGA supplementation took one placebo capsule per day for three months instead.

**Table 2:** Composition per capsule of omega-3 supplement (ROMEGA Actric Herring Caviar Oil, Arctic Nutrition AS, Ørsta, Norway)

Nutrient	Amount
Total omega-3	235 mg
Phospholipid omega-3	170 mg
DHA	160 mg
EPA	50 mg
Choline	21 mg
Vitamin D	500 mg
Vitamin E	1 mg

All the ophthalmological assessment was reevaluated at three months. In order to improve compliance and ensure the validity of the study result, all patients were required to deliver the empty boxes of supplementation received.

The primary outcomes were visual function indexes including mean deviation (MD) and pattern standard deviation (PSD) on the VF test. Secondary outcomes were IOP, BCVA, RNFLT, and adverse events. The following criteria were established to exclude subjects: (1) IOP > 18 mm Hg detected; (2) An intercurrent ocular or neurologic disease which might affect the VF and OCT results; (3) the patient moved, voluntary withdrew, died, or could not accomplish the treatment or follow-up. The safety outcomes and any adverse events were evaluated during the whole period of the study.

### Statistical analysis

Data were analyzed using SPSS version 23.0 (SPSS Inc., Chicago, IL, USA) and GraphPad Prism (version 9.0; GraphPad Inc., USA). As it was a preliminary study to estimate the effect of omega-3 supplementation from herring caviar oil in IOP-controlled POAG patients, no similar study is available in the literature and no effect size can be obtained a priori. Therefore, the sample size was designed based on feasibility. The normality of parameters was assessed by a Kolmogorov–Smirnov test. When the parameters were in accordance with normal distribution, a paired or unpaired t-test was conducted. Mann–Whitney *U*-test or Wilcoxon matched-pairs signed-rank test was performed when the data did not follow the normal distribution. Fisher’s exact test was conducted to compare the ratio difference between groups. The summarized data were expressed as mean ± SD. *P* < 0.05 was considered statistically significant.

## Results

Sixty-five Chinese POAG patients were initially enrolled and all patients showed non-profession on the current intervention. Nine patients were excluded due to their medical history indicating a 24-h peak IOP lesser than 21 mm Hg without antiglaucomatous medications, which supported the diagnosis of normal tension glaucoma (NTG). During the follow-up, three patients had IOP elevated due to irregular ocular hypotensive medication, and three patients were lost to follow-up. No patient was excluded for visual acuity decreased due to the sudden exacerbation of cataracts or other ocular diseases that could affect the VF and OCT assessment. Thus, a total of 50 eyes of 50 patients were included for analysis, including 36 males and 14 females. The average age was 49.40 ± 10.47 years, ranging from 30 to 68 years. The baseline clinical characteristics of control group and intervention group were shown in Table 3, and no differences were detected between groups.

**Table 3:** Baseline clinical characteristics of 50 enrolled eyes

	Control group (*n* = 31) mean (SD)	Intervention group (*n* = 19) mean (SD)	*P* value
Sex, male/female	21/10	15/4	0.52**
Age (years)	48.88 (10.53)	52.95 (8.85)	0.21*
BCVA	0.75 (0.22)	0.73 (0.29)	0.93*
IOP (mmHg)	13.73 (2.12)	13.88 (2.51)	0.82*
Antiglaucoma medications (numbers)	1.55 (0.62)	1.90 (0.81)	0.15*
MD (dB)	-16.61 (5.21)	-16.11 (4.14)	0.72#
PSD (dB)	12.51 (2.62)	13.23 (1.15)	0.26#
AGIS score	10.81 (3.43)	12.26 (2.79)	0.13#
RNFLT (μm)	59.10 (8.53)	56.05 (9.91)	0.21*
Glaucoma stage (moderate/advanced)	5/26	4/15	0.72**

*BCVA* best-corrected visual acuity, *IOP* intraocular pressure, *MD* median deviation, *PSD* pattern standard deviation, *RNFLT* retinal nerve fiber layer thickness, *AGIS score* advanced glaucoma intervention study visual defect score

^*^Mann–Whitney U-test

^#^Unpaired t-test

^**^Fisher’s exact test

**Fig. 1:**
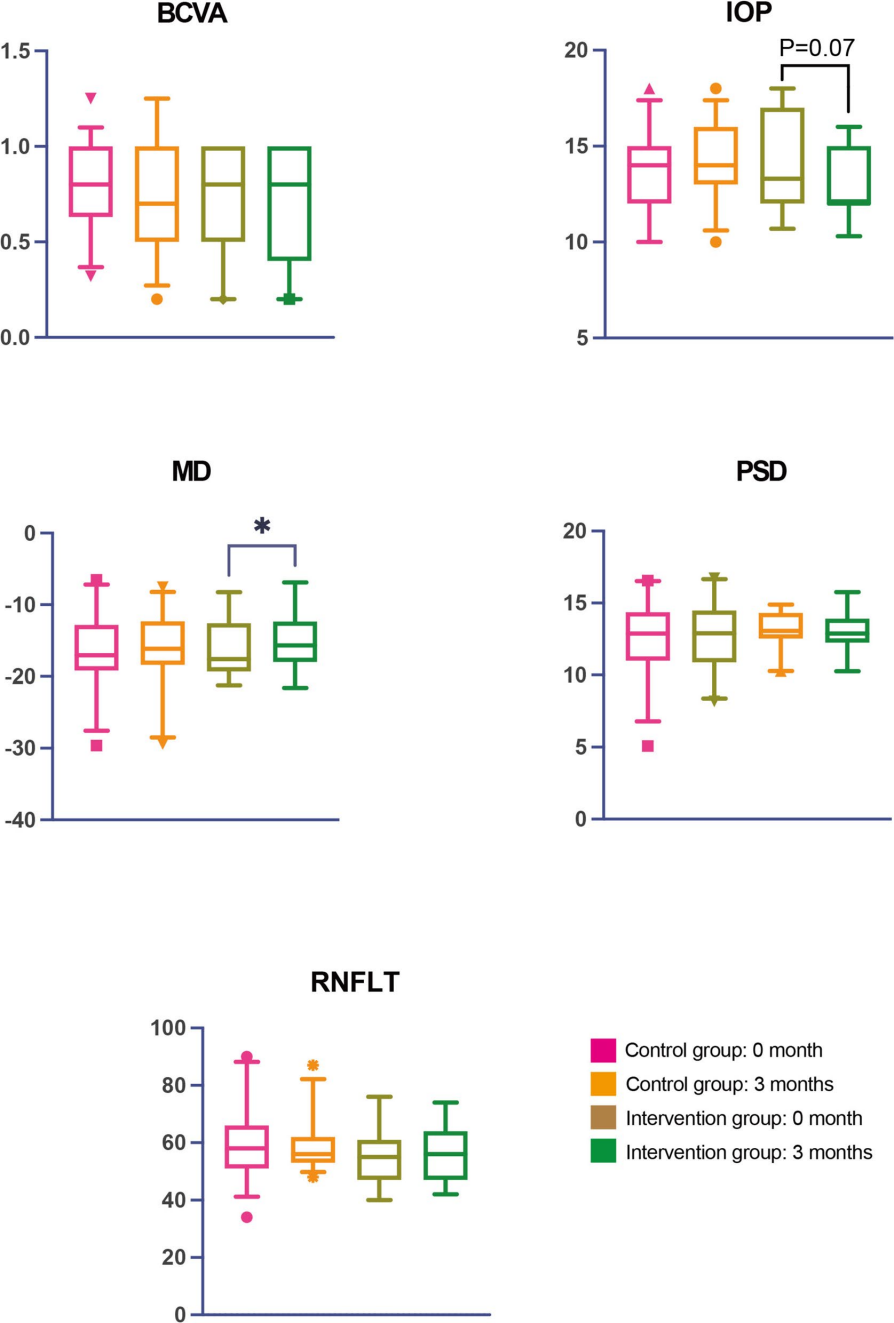
The values of BCVA, IOP, MD, PSD, RNFLT at baseline and 3 months in the control group and intervention group. BCVA: best-corrected visual acuity, IOP: intraocular pressure, MD: median deviation, PSD: pattern standard deviation, RNFLT: retinal nerve fiber layer thickness. * indicates *p* < 0.05

**Fig. 2:**
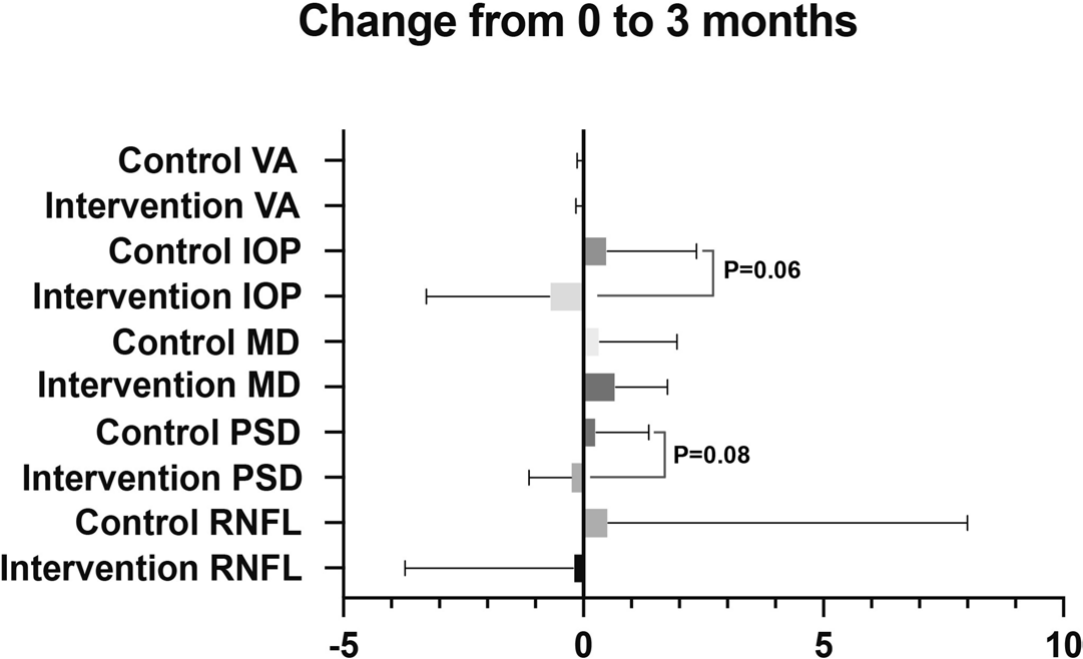
Changes in the evaluated variables from baseline in the control group and the omega-3 supplements intervention group. BCVA: best-corrected visual acuity, IOP: intraocular pressure, MD: median deviation, PSD: pattern standard deviation, RNFLT: retinal nerve fiber layer thickness

Table 4 illustrated the comparison of clinical characteristics of the control group and the intervention group at 3 months and the variables change from baseline. The medication prescriptions and the number of antiglaucomatous medications for all patients remained unchanged during the follow-up period. Upon comparing the variables at baseline and 3-month follow-up, we found that only the MD value of intervention group showed significantly improvement, increasing from -16.11 ± 4.14 dB at baseline to -15.44 ± 4.10 dB at three months (paired t-test, *p* = 0.01), with a change of 0.67 ± 1.08 dB [95% CI 0.15–1.20] (compared with the change of control group by unpaired t-test, *p* = 0.42) (Figs. 1, 2). In contrast, the control group's MD value did not show a significant change (from 16.61 ± 5.21 dB to -16.28 ± 5.21 dB, Mann–Whitney U-test, *p* = 0.32). The mean PSD also demonstrated an improved performance after omega-3 supplementation intervention, decreasing from 13.23 ± 1.15 dB to 12.97 ± 1.34 dB (paired t-test, *p* = 0.22), with a change of -0.25 ± 0.88 dB [95% CI -0.68–0.17] (compared with the change of control group by unpaired t-test, *p* = 0.08), however, the comparison had no statistically significant difference (Figs. 1, 2). Additionally, the comparison of MD and PSD at 3-month follow-up between groups revealed no statistically significant difference.

**Table 4:** Comparison of clinical characteristics of control group and intervention group at 3 months

	3-month follow-up					Change from 0 to 3 months		
	Control group mean (SD)	*P* value'	Intervention group mean (SD)	*P* value'	*P* value''	Control group mean (SD) [95 CI%]	Intervention group mean (SD) [95 CI%]	*P* value
BCVA	0.72 (0.26)	0.27*	0.70 (0.27)	0.15*	0.99**	-0.02 (0.11) [-0.06, 0.02]	-0.03 (0.12) [-0.09, 0.02]	0.91**
IOP (mmHg)	14.22 (2.04)	0.15#	13.17 (1.67)	0.20*	0.07**	0.49 (1.87) [-0.20, 1.18]	-0.71 (2.57) [-1.94, 0.53]	0.06##
MD (dB)	-16.28 (5.21)	0.32*	-15.44 (4.10)	0.01#	0.93**	0.33 (1.62) [-0.26, 0.93]	0.67 (1.08) [0.15, 1.20]	0.42##
PSD (dB)	12.77 (2.26)	0.18#	12.97 (1.34)	0.22#	0.73##	0.27 (1.09) [-0.13, 0.67]	-0.25 (0.88) [-0.68, 0.17]	0.08##
RNFLT (μm)	59.61 (12.21)	0.81*	55.84 (10.78)	0.99*	0.23**	0.52 (7.48) [-2.23, 3.26]	-0.21 (3.51) [-1.91, 1.48]	0.99**

*CI* confidence interval, *BCVA* best-corrected visual acuity, *IOP* intraocular pressure, *MD* median deviation, *PSD* pattern standard deviation, *RNFLT* retinal nerve fiber layer thickness

'Comparision the changes before and after 3 months within one group

''Comparison the variables between control and intervention group at 3 months

^*^Wilcoxon matched-pairs signed rank test

^**^Mann–Whitney U-test

^#^Paired t-test

^##^Unpaired t-test

Mean IOP slightly decreased in the omega-3 supplementation group, from 13.88 ± 2.51 mm Hg baseline to 13.17 ± 1.67 mm Hg at three months (Mann–Whitney U-test, *p* = 0.07), representing with a change of − 0.71 ± 2.57 mm Hg [95% CI − 1.94–0.53] (compared with the change of control group by unpaired t-test, p = 0.06) with the same prescription of IOP-lowering medicines (Figs. 1, 2). The BCVA and RNFLT remained stable during the 3-month follow-up in all 50 eyes (Fig. 1). Furthermore, no adverse effect attributable to omega-3 supplementation was notified during the observation.

## Discussion

In this 3-month preliminary study, we observed that oral omega-3 fatty acids supplementation from phospholipid-rich herring caviar oil was safe and might be beneficial for POAG patients.

Omega-3 fatty acids is proven to be beneficial in many neurodegenerative disorders, such as age-related macular dystrophy, diabetic retinopathy, myopia, and Alzheimer’s disease (AD) [6, 8, 19, 20]. In the aspect of glaucoma, clinical studies suggest that a lack of dietary omega-3 fatty acids is associated with a high risk of primary POAG [21–23], while oral omega-3 fatty acids supplementation could reduce the IOP in normotensive persons [24, 25] as well as decrease oxidative stress and inflammation in pseudoexfoliative glaucoma [15]. Research on experimental glaucoma models demonstrates that omega-3 fatty acids can lower IOP by increasing aqueous humor outflow facility, suppress retinal inflammation, and enhance RGCs survival [9, 10, 12, 26]. Nonetheless, there are only a few reports in the literature regarding the effect of oral omega-3 fatty acids supplementation on the visual function of patients with POAG.

ROMEGA herring caviar oil contains 34% of its omega-3 fatty acids in phospholipid form [27], with a DHA: EPA ratio of about 3:1. Omega-3 fatty acids in phospholipid form can cross the blood-retinal barrier and retinal pigment epithelium (RPE) via the transporter Mfsd2a directly [28, 29] reaching a high concentration in the eyeball, whereas omega-3 fatty acids in triglyceride form, free fatty acid form, or ethyl ester form in most fish oil supplementations cross the barrier via passive diffusion which decreases the utilization [19].

To minimize the learning effects and variability of the VF test, we enrolled pre-diagnostic POAG patients who had undergone this examination more than three times and excluded the unreliable results. Among the comparison of MD, PSD, BCVA, IOP, and RNFLT within and between groups, only the MD value showed significantly increase in the intervention group. The change of PSD exhibited a slightly decrease between the control and the intervention group but did not reach the significant differences. These results indicate that intake of ROMEGA supplementation might be benefit for the visual function improvement of IOP-control POAG patients. The possible neuroprotective mechanisms are as follows: Firstly, omega-3 fatty acids in ROMEGA containing antioxidants may facilitate free radical scavenging and impeding inflammatory immune response in glaucomatous degeneration [9–11, 15]. Secondly, The fatty acids are also beneficial in regulating blood circulation by serving as precursors of prostaglandins, suggesting their therapeutic effect on POAG with impaired blood flow [22, 24]. Furthermore, as it has been shown that omega-3 fatty acids have the ability to act as neurotrophic factors in many neurodegenerative disorders [20, 30–32], dietary omega-3 fatty acids intake might stimulate and modulate the expression of brain-derived neurotrophic factor (BDNF) and glial cell-derived neurotrophic factor (GDNF) to promote neuronal survival in the retina[16, 20, 33] for protecting against glaucoma.

The IOP was a potential confounder in this preliminary evaluation, presenting a reduction of 0.71 ± 2.57 mm Hg with a *p* value of 0.06. Downie et al. supplemented 72 normotensive adults with a dosage of about 500 mg/day DHA + 1000 mg/day EPA for 3 months, which was nearly four times as much as we did, and finally found a minor and significant reduction of 0.6 ± 0.2 mm Hg in IOP[24]. In a omega-3 supplementation evaluation for glaucoma patients with dry eye, patients were instructed to intake the supplementation containing a total dose of 1050 mg/day TG-DHA + 127.5 mg/day EPA for 12 weeks, which decreased the IOP from 16.4–16.5 mm Hg to 16.1 mm Hg. One capsule of ROMEGA contains 160 mg of DHA and 50 mg of EPA. As the number and prescription of antiglaucomatous remained unchanged in all the enrolled patients during the observation period, it is speculated that the reduction effect on IOP may be dose-dependent, and that the visual function improvement might be partially resulted from the change of IOP.

There are some limitations of our study. Firstly, the sample size was relatively small, and the 3-month observation period was not long enough to obtain a well-documented conclusion about VF progression. Secondly, we set an IOP of ≤ 18 mm Hg to define controlled IOP, which may be an arbitrary limit as each POAG patient has their own personalized target IOP. Thirdly, the majority of POAG patients included in our study were in the advanced stage with poor VF performance and thin RNFLT, limiting the evaluation of neuroprotective effect on RGCs.

Given the exploratory nature of our preliminary study, it primarily assessed feasibility and the potential effects of the intervention. The sample size was determined based on resource and time constraints. However, this sample size was sufficient to evaluate study processes and generate initial effect estimates, which could inform future the design of randomized clinical trials. Future studies can be conducted by increasing sample size, extending the follow-up period to at least one year with five VF measurements, adding a contrast sensitivity test, increasing dosage and investigating the dosage-dependent effect, detecting the content of omega-3 fatty acids in plasma, aqueous humor as well as vitreous humor. A standard and comprehensive statistical analysis of sample size will also be undertaken for the main study based on the results of current preliminary study. Additionally, optical coherence tomography angiography for evaluating retinal blood flow and neuroprotective biomarkers (e.g., BDNF and GDNF) will also be assessed to provide a more comprehensive evaluation of the effects of omega-3 supplementation. Since AD shares some of the same pathogenesis with POAG [34], ROMEGA supplementations could also be applied in patients with AD to evaluate its neuroprotective effect.

In summary, our study shows that oral omega-3 fatty acids supplementation from herring caviar oil for three months improved visual function without harmful effects in IOP-controlled POAG patients, and its neuroprotective potential in neurodegenerative disease warrants larger trials to justify.

## Data Availability

No datasets were generated or analysed during the current study.

## Abbreviations

POAG: Primary open-angle glaucoma
IOP: Intraocular pressure
RGCs: Retinal ganglion cells
VF: Visual field
MD: Mean deviation
VFI: Visual field index
PSD: Pattern standard deviation
AGIS: Advanced glaucoma intervention study
OCT: Optical coherence tomography
RNFLT: Retinal nerve fiber layer thickness
AD: Alzheimer’s disease
